# No sonographer, no radiologist: New system for automatic prenatal detection of fetal biometry, fetal presentation, and placental location

**DOI:** 10.1371/journal.pone.0262107

**Published:** 2022-02-09

**Authors:** Junior Arroyo, Thomas J. Marini, Ana C. Saavedra, Marika Toscano, Timothy M. Baran, Kathryn Drennan, Ann Dozier, Yu Tina Zhao, Miguel Egoavil, Lorena Tamayo, Berta Ramos, Benjamin Castaneda

**Affiliations:** 1 Laboratorio de Imágenes Médicas, Departamento de Ingeniería, Pontificia Universidad Católica del Perú, Lima, Peru; 2 Department of Imaging Sciences, University of Rochester Medical Center, Rochester, New York, United States of America; 3 Department of Obstetrics and Gynecology, University of Rochester Medical Center, Rochester, New York, United States of America; 4 Department of Public Health, University of Rochester Medical Center, Rochester, New York, United States of America; 5 Research & Development, Medical Innovation & Technology, Lima, Perú; University of North Carolina at Chapel Hill, UNITED STATES

## Abstract

Ultrasound imaging is a vital component of high-quality Obstetric care. In rural and under-resourced communities, the scarcity of ultrasound imaging results in a considerable gap in the healthcare of pregnant mothers. To increase access to ultrasound in these communities, we developed a new automated diagnostic framework operated without an experienced sonographer or interpreting provider for assessment of fetal biometric measurements, fetal presentation, and placental position. This approach involves the use of a standardized volume sweep imaging (VSI) protocol based solely on external body landmarks to obtain imaging without an experienced sonographer and application of a deep learning algorithm (U-Net) for diagnostic assessment without a radiologist. Obstetric VSI ultrasound examinations were performed in Peru by an ultrasound operator with no previous ultrasound experience who underwent 8 hours of training on a standard protocol. The U-Net was trained to automatically segment the fetal head and placental location from the VSI ultrasound acquisitions to subsequently evaluate fetal biometry, fetal presentation, and placental position. In comparison to diagnostic interpretation of VSI acquisitions by a specialist, the U-Net model showed 100% agreement for fetal presentation (Cohen’s κ 1 (p<0.0001)) and 76.7% agreement for placental location (Cohen’s κ 0.59 (p<0.0001)). This corresponded to 100% sensitivity and specificity for fetal presentation and 87.5% sensitivity and 85.7% specificity for anterior placental location. The method also achieved a low relative error of 5.6% for biparietal diameter and 7.9% for head circumference. Biometry measurements corresponded to estimated gestational age within 2 weeks of those assigned by standard of care examination with up to 89% accuracy. This system could be deployed in rural and underserved areas to provide vital information about a pregnancy without a trained sonographer or interpreting provider. The resulting increased access to ultrasound imaging and diagnosis could improve disparities in healthcare delivery in under-resourced areas.

## Introduction

**Fig 1:**
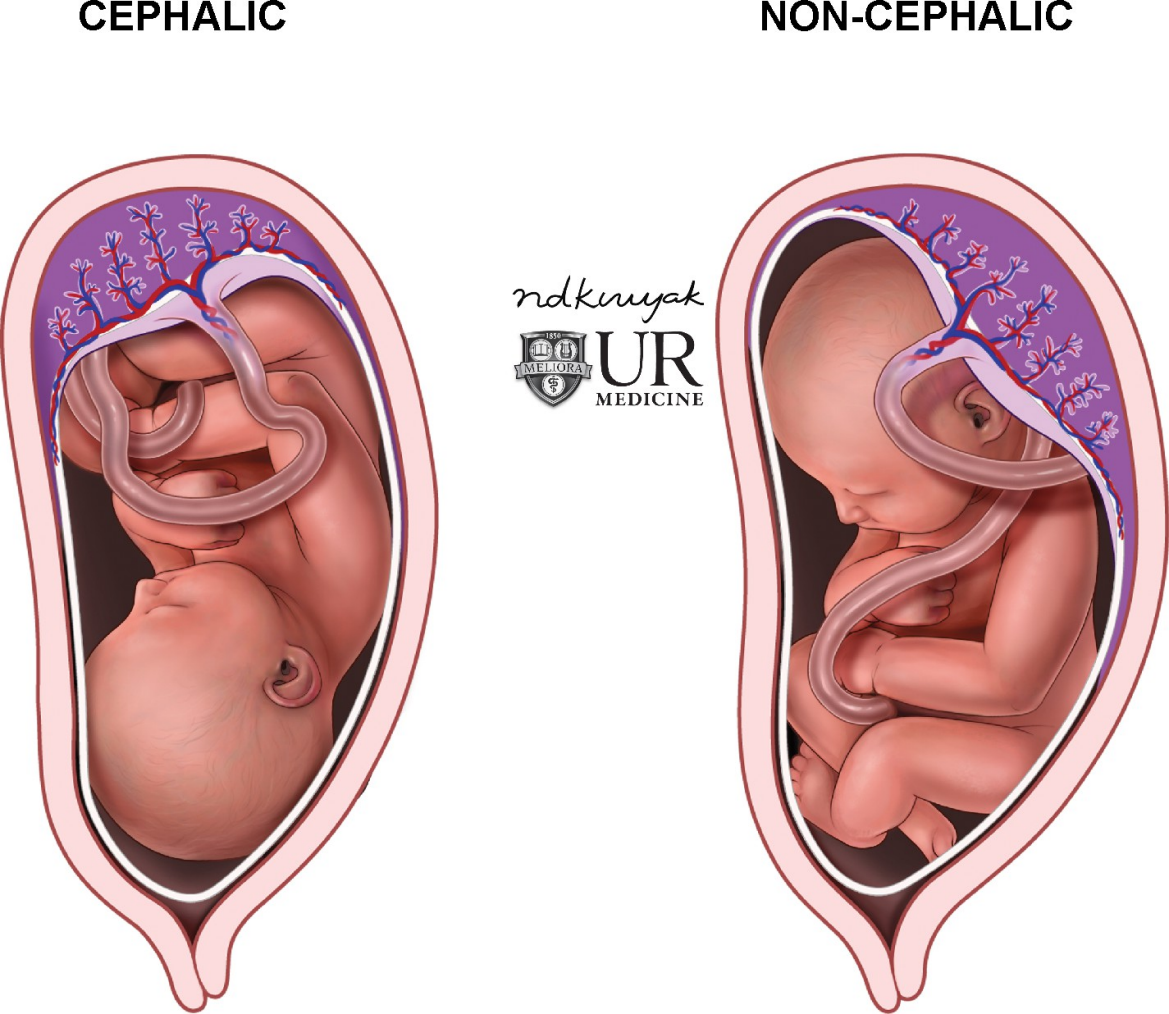
Fetal positions. Illustration of cephalic and non-cephalic fetal positions. Non-cephalic fetal presentations are important to identify prior to delivery.

**Fig 2:**
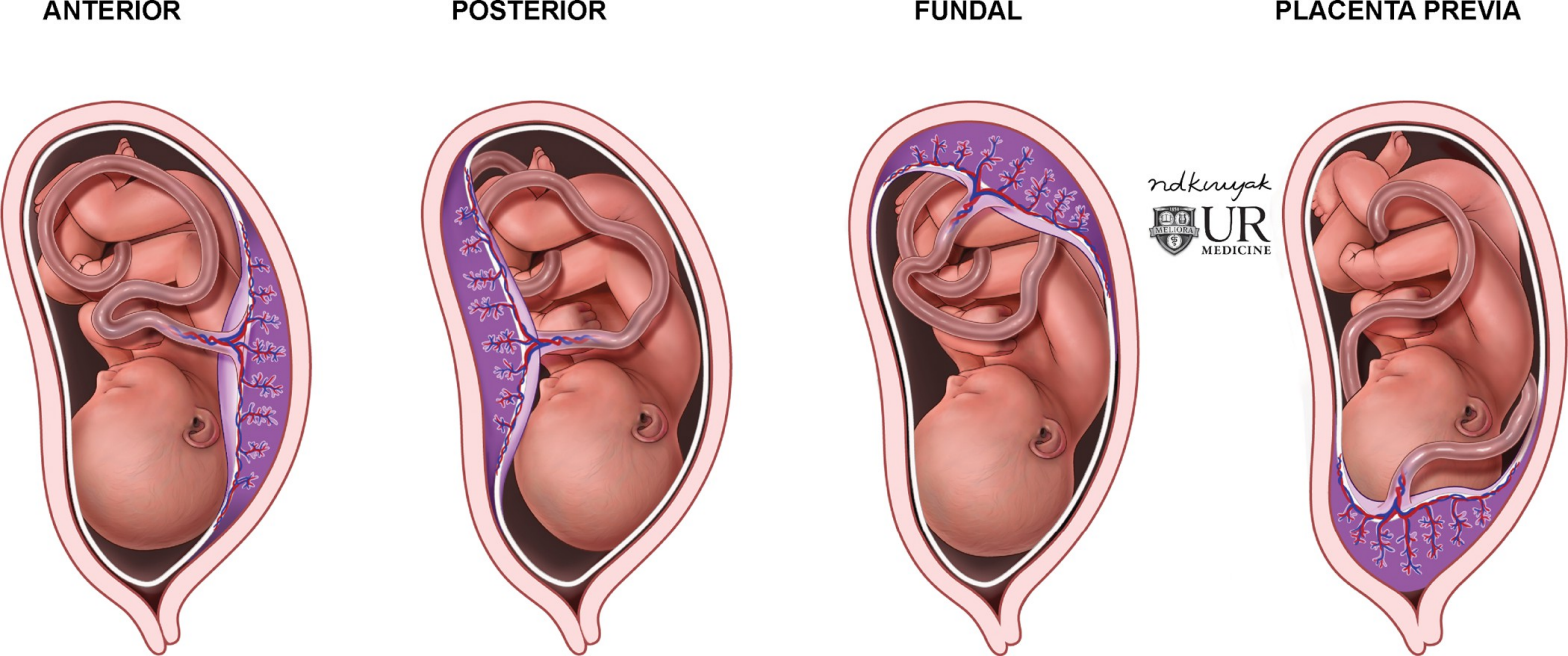
Placental positions. Illustration of different placental positions. The fundal position straddles anterior and posterior positions. Fundal, anterior, and posterior placental positions have no significant clinical impact. However, they are vital to distinguish from placenta previa which can be life-threatening.

**Fig 3:**
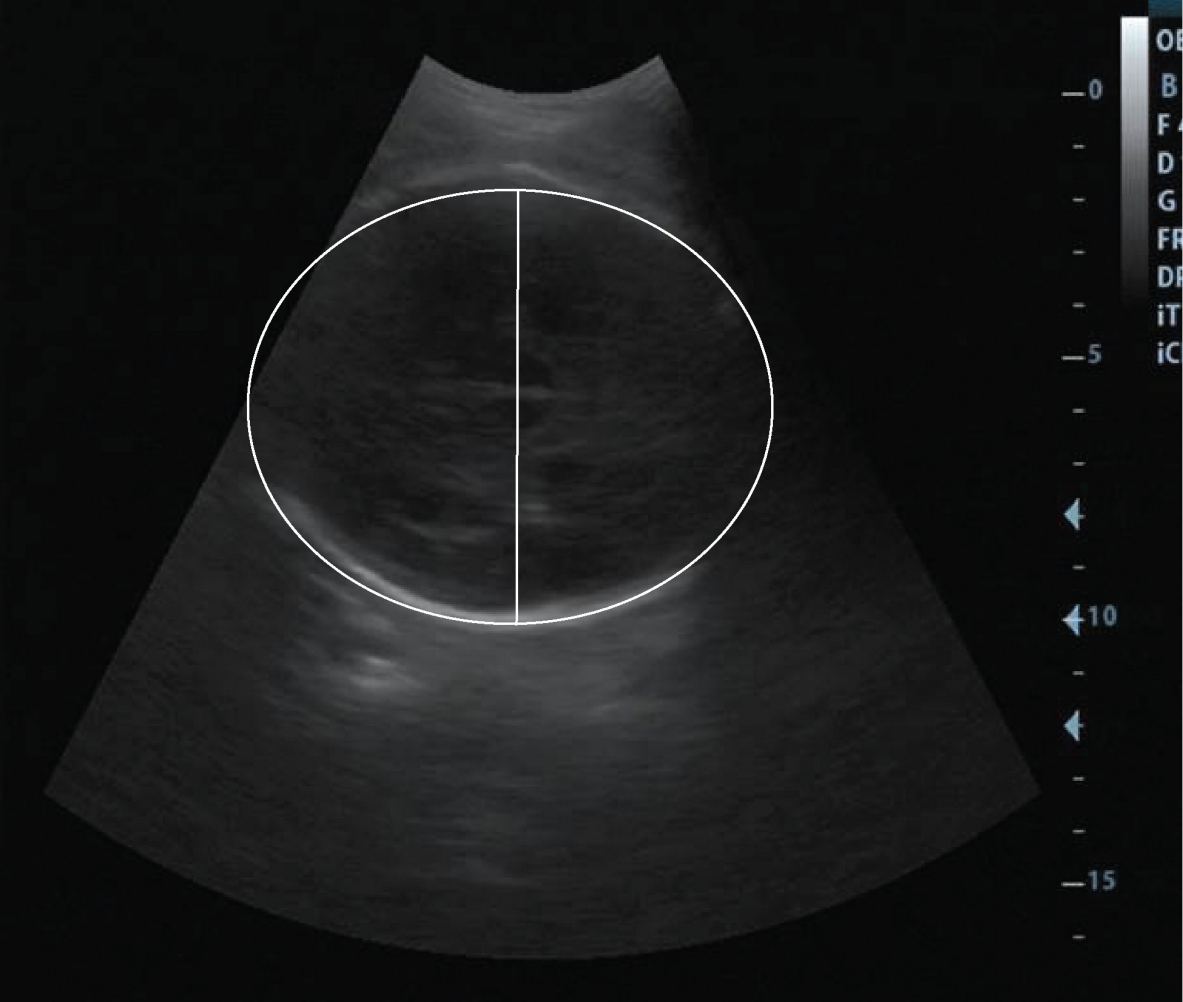
Biometry measurements of the head circumference and biparietal diameter. Head circumference (ellipse) and biparietal diameter (line) measurements are illustrated.

Ultrasound remains a vital component of antenatal care, allowing for evaluation of the fetal presentation, fetal number, placental location, and fetal biometry [1–3]. However, for millions in rural and underserved areas, there is limited access to ultrasound imaging, leading to potentially preventable harm from associated pregnancy complications [4–6]. Increased detection of these pregnancy complications through ultrasound can allow for appropriate referral for delivery care in more resourced centers with trained providers. We propose that this barrier to ultrasound access may be overcome in a locally sustainable and resource-conscious way through the use of standardized scanning protocols combined with artificial intelligence obviating the need for an interpreting provider and an experienced sonographer. Prior studies demonstrate that individuals without prior ultrasound training (non-specialists) can obtain diagnostic imaging for placental position, fetal presentation and number, and amniotic fluid volume using volume sweep imaging (VSI) [7, 8]. Different fetal and placenta positions and their significance are illustrated and described in Figs 1 and 2. Measurements of fetal biometry are also often possible through this approach including evaluation of head circumference (HC) and biparietal diameter (BPD) (Fig 3).

**Fig 4:**
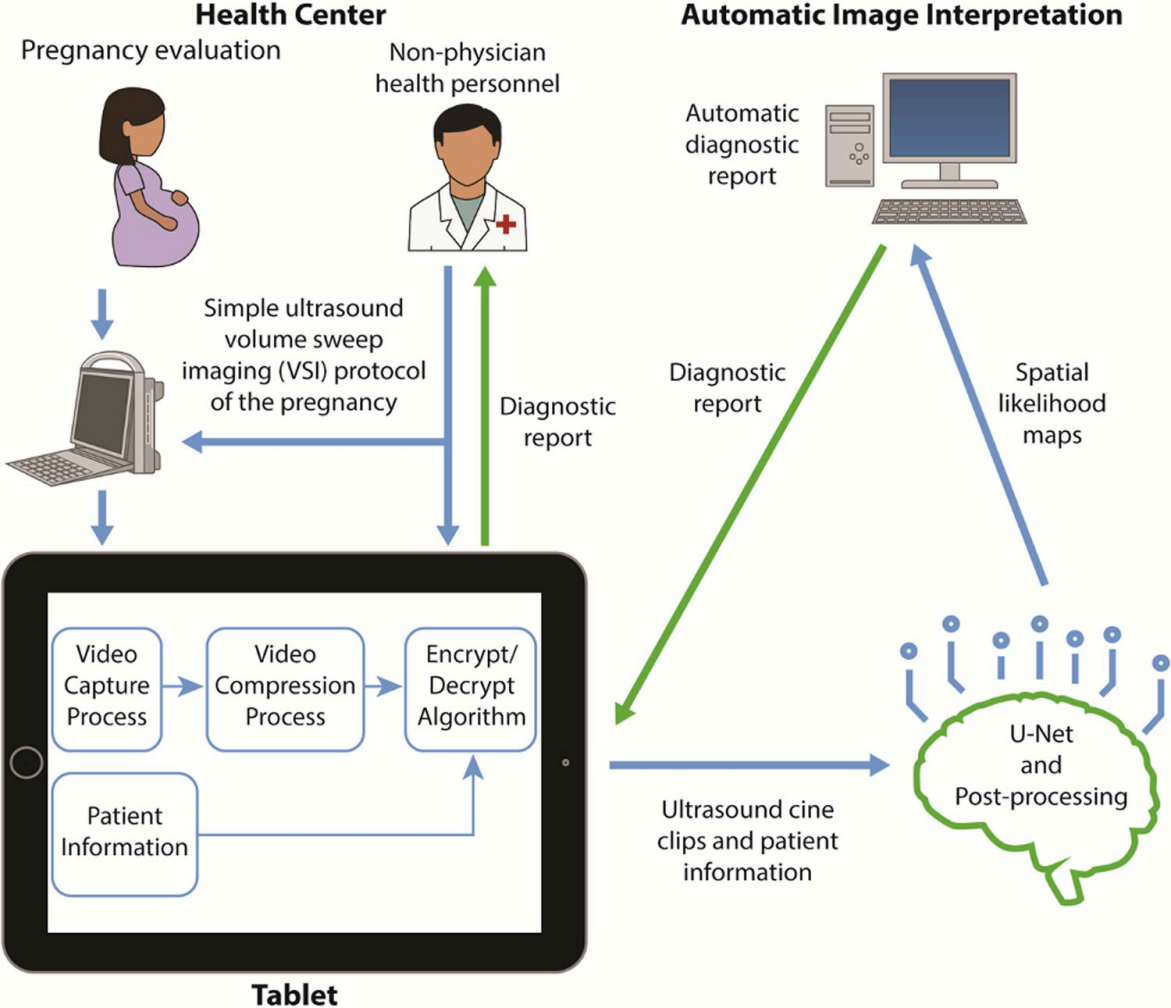
Proposed system for automatic obstetric diagnosis without an experienced sonographer or radiologist. Schematic diagram shows utilization of VSI combined with U-Net for rapid automatic image interpretation. The blue arrows signify the input of information into the automatic diagnostic framework. The green arrows signify the output of information.

VSI is an imaging technique in which an operator sweeps the ultrasound probe over the target region to obtain a full volumetric acquisition of the target region [7]. Sweeps are standardized and based solely on external body landmarks requiring no significant technical skills or knowledge of anatomy. Each sweep is saved as a cine clip which is then interpreted by a specialist. While this approach removes the need for a trained sonographer, a trained medical provider is still required for interpretation. Building upon this, we sought to test whether artificial intelligence and deep learning may be a strategy that can additionally eliminate the need for a trained medical provider for study interpretation, thereby reducing the resource burden of this technologic innovation for application in rural and under-resourced areas. Fig 4 demonstrates the proposed system. In these settings, the alternative to artificial-intelligence assisted diagnosis would be traveling to a health-clinic with ultrasound services, a VSI Obstetric ultrasound interpreted by an expert, or no imaging at all.

Deep learning is a representation learning approach, which is able to extract mid-level and high-level features from images to perform automatic image analysis, such as detection, segmentation, and classification [9]. Fetal biometry requires standardized imaging planes that require technical skill and anatomic knowledge to obtain. Fully convolutional neural networks have been previously used successfully for analyzing 2D ultrasound images associated with fetal structures, but these rely on an imaging expert to find the structure and plane of interest [10]. Once the standard planes are obtained, studies have shown that identification of disease states like congenital heart conditions is possible with artificial intelligence [11–13]. Given the difficulty in identifying the appropriate scan planes, other attempts have been made to identify these fetal landmarks in live video to guide the operator into identifying the plane of interest [14, 15]. While these approaches assist in a complex task, they would still require a somewhat experienced sonographer to obtain the images, which limits deployment to under-resourced areas. Finally, 3D ultrasound is an option to provide more detailed information than 2D approaches regarding the spatial location of organs and structures [9]. However, 3D ultrasound is more expensive in terms of equipment and computational cost for deep learning implementation. It is therefore unsuitable for application in under-resourced areas at this time.

The use of deep learning combined with standardized VSI imaging acquisitions offers a new possibility for determination of spatial location of fetal structures using 2D ultrasound. Such an approach would require neither a specialist nor an experienced sonographer (Fig 4). This combination has been previously shown to successfully measure the fetal head circumference using a VGG-Net and U-Net from images acquired using VSI and also detect placenta position by using a U-Net-inspired network from images acquired using VSI [16, 17]. In this study, we developed an automatic system based on the U-Net and VSI acquisitions obtained at a clinic in Peru to identify fetal presentation, placental location, and assess fetal head biometry. Segmentation with deep learning and a score-based algorithm were used to identify the fetal presentation and placental position. Head biometry was determined using head segmentation masks with post-processing algorithms. In addition, the diagnostic capacity of the automatic system was assessed by evaluating data from a hold-out test set, which were not used in the initial U-Net training. Comparison was made both with the VSI acquisitions interpreted by an Obstetrician and standard of care imaging obtained and interpreted by a radiologist.

## Materials and methods

### Participants

Participants were 58 third trimester pregnant women presenting for routine prenatal ultrasound at the Conde de la Vega Health Center in Lima, Peru between October 2018 and March 2019. The average age of participants was 25.8 years with a standard deviation of 6 years and a range of 18–41 years of age. All participants underwent VSI ultrasound performed according to the specifications below. Subjects were recruited as a non-random convenience sample and were included if over age 18. There were no exclusion criteria. Because this was a pilot study and the data had already been collected, a priori sample size was not calculated. All participants were informed about the procedure and provided written informed consent. This study was authorized by the Institutional Review Board at the Hospital Nacional Docente Madre Nino San Bartolome, and all the participants provided written informed consent.

### Volume sweep imaging

**Fig 5:**
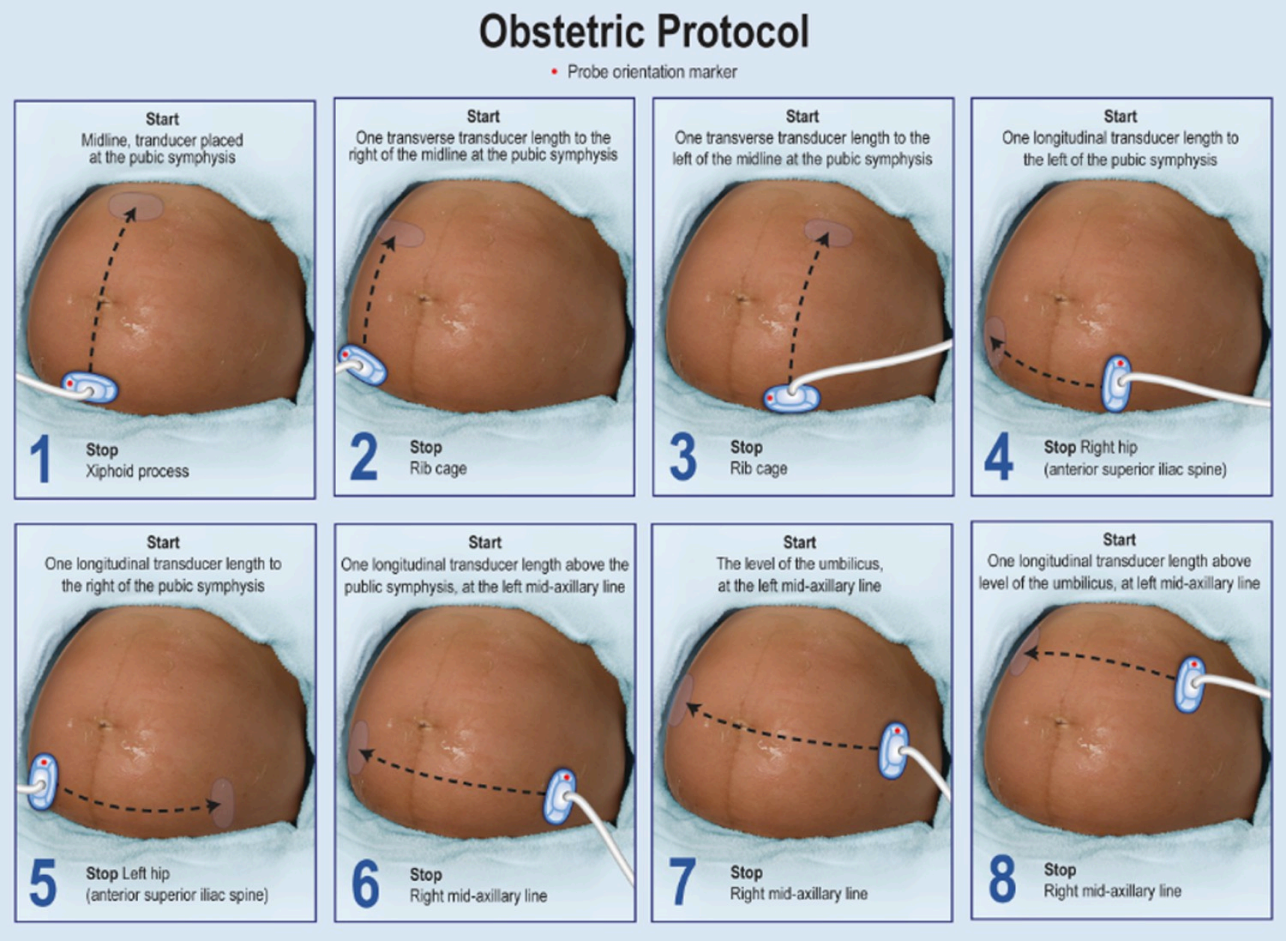
Obstetric ultrasound volume sweep imaging protocol. Poster depicting each step in the VSI protocol.

VSI is an imaging technique that was developed to increase access to ultrasound in underserved areas. In VSI, the operator requires no significant medical background or technical ultrasound skill. Individuals have been shown to learn VSI after a few hours of training, and no significant ultrasound experience or anatomical knowledge is required [7, 8, 18–20]. A VSI protocol consists of a series of ultrasound probe sweeps over the target region demarcated by easily recognized external body landmarks. The operator is not interpreting the images and is instructed to focus on the probe position instead of looking at the ultrasound screen. Each sweep of the probe is saved as a cine clip which can be then interpreted by a specialist or deep learning algorithm. VSI has been successfully integrated into existing telemedicine infrastructure in Peru [7]. The Obstetric VSI examination is shown in Fig 5. This VSI protocol is based on eight sweeps of the ultrasound probe which constitute a full volumetric examination of the pregnant abdomen. Obstetric VSI has previously demonstrated excellent agreement with standard of care ultrasound performed by a radiologist [8].

### Test methods

The primary reference test in this study was the physician specialist interpretation of VSI scans for descriptive characteristics and quantitative measurements including fetal presentation, placental location, and fetal biometric parameters for the 58 pregnant women. All fetal measurements were obtained using standard guidelines [3]. This primary reference standard was chosen as opposed to standard of care ultrasound to isolate variables as the goal of this paper was to evaluate the artificial intelligence algorithm derived from the VSI scans. Nonetheless, as standard of care ultrasound would be considered the ultimate ground truth for both VSI and artificial intelligence, we included these results as a second reference standard for consideration. Previously VSI has shown statistically significant agreement with standard of care ultrasound in this same sample of patients [8]. The standard of care ultrasound exams used as a reference standard were performed and interpreted concurrently at the time of the VSI scan by an expert radiologist. This diagnostic examination was conducted in accordance with standardized guidelines [1]. The radiologist’s images from this separate examination were not used in any artificial intelligence analysis. All imaging was performed using a portable Mindray DP-10 ultrasound scanner (Mindray, China) with a 4.5 MHz transducer. An individual without prior ultrasound experience acquired the VSI images after 8 hours of training and was blinded to the results of the standard of care ultrasound. Similarly, the radiologist performing and interpreting the standard of care ultrasound was blinded to the results of the VSI scan. Additionally, the Obstetrician interpreting the VSI scans was not given access to the clinical history.

### Training, validation, and testing

The automatic system development was performed with the VSI acquisitions of the first 30 sequentially recruited participants using a leave-one-out approach described below. The entire 58 patient sample was not used in the leave-one-out cross-validation due to the labor-intensive process of segmentation and limitations on access to the computational power for higher size samples. From training, validation, and testing of these 30 patients, a file containing optimized training weights from the U-Net network was obtained, allowing automated direct and rapid diagnosis for novel data. The subsequent 28 recruited patients (not used in the initial leave-one-out cross-validation) were analyzed using the weights file (hold-out test phase). There were no differences in the patient population between the different samples. This hold-out test set was used to determine the performance of the system when facing novel patients as if this automatic system were implemented in clinical practice.

### Leave-one-out cross-validation

#### Data preparation

**Fig 6:**
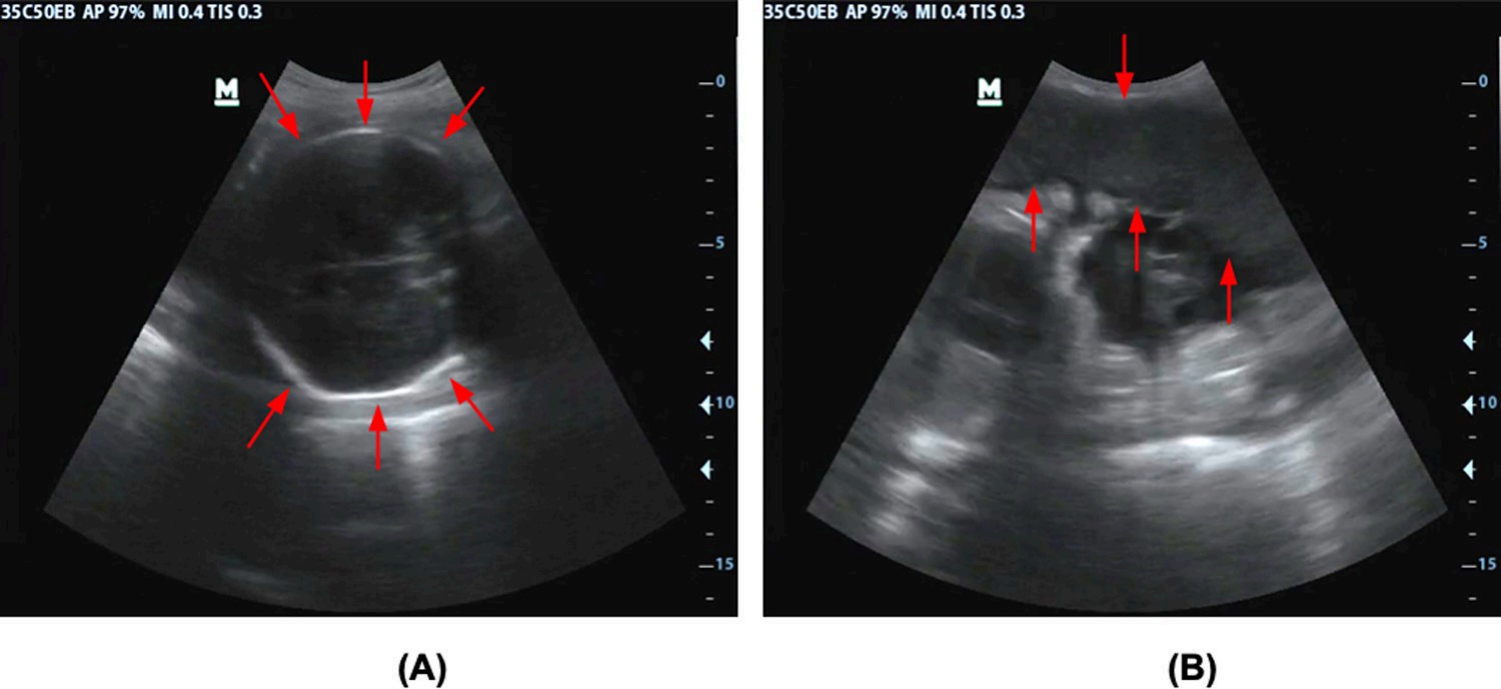
Representative 2D ultrasound images from VSI acquisition. Red arrows identify the head (A) and placenta (B).

The 2D ultrasound images from VSI exams were exported in MP4 cine clips or DICOM format and then converted from these formats to TIFF format using ImageJ software (National Institutes of Health, Bethesda, MD). Preprocessing stages including image conversion from RGB to grayscale, image cropping of the scan to avoid set-up parameters of the device, and image resizing to 128×128 pixels were performed in MATLAB 2019b (Mathworks, Natick, Massachusetts, USA). Ground truth segmentation of the fetal head and placenta was performed by a single radiologist in 30 subjects to produce binary masks used in training. Fig 6 depicts the region of interest for this study.

#### Network architecture and parameters

The architecture of the deep learning network used in this study was the U-Net [21]. It consists of contracting and expansive paths and is composed of 23 convolutional layers. A downsampled version of the ultrasound images (size of 128×128) and their corresponding segmentation masks were used as the input data of the network. The weight parameters were initialized using He weight initialization [22]. As in the original U-Net, the number of filters is doubled after each downsampling operation and halved after each upsampling operation. Batch normalization was introduced to reduce internal covariate shift and to accelerate training [23]. A rectified linear unit (ReLU) was used after each convolutional layer. Additionally, a 2×2 max-pooling layer was used on the downsampling side. Finally, the output layer was activated with a sigmoid function. A batch size of 16 frames was used for all experiments. The Adam optimizer was used to control the binary cross-entropy loss, and both the Dice loss and Jaccard distance assessed convergence [24].

#### Training using the leave-one-out approach

**Fig 7:**
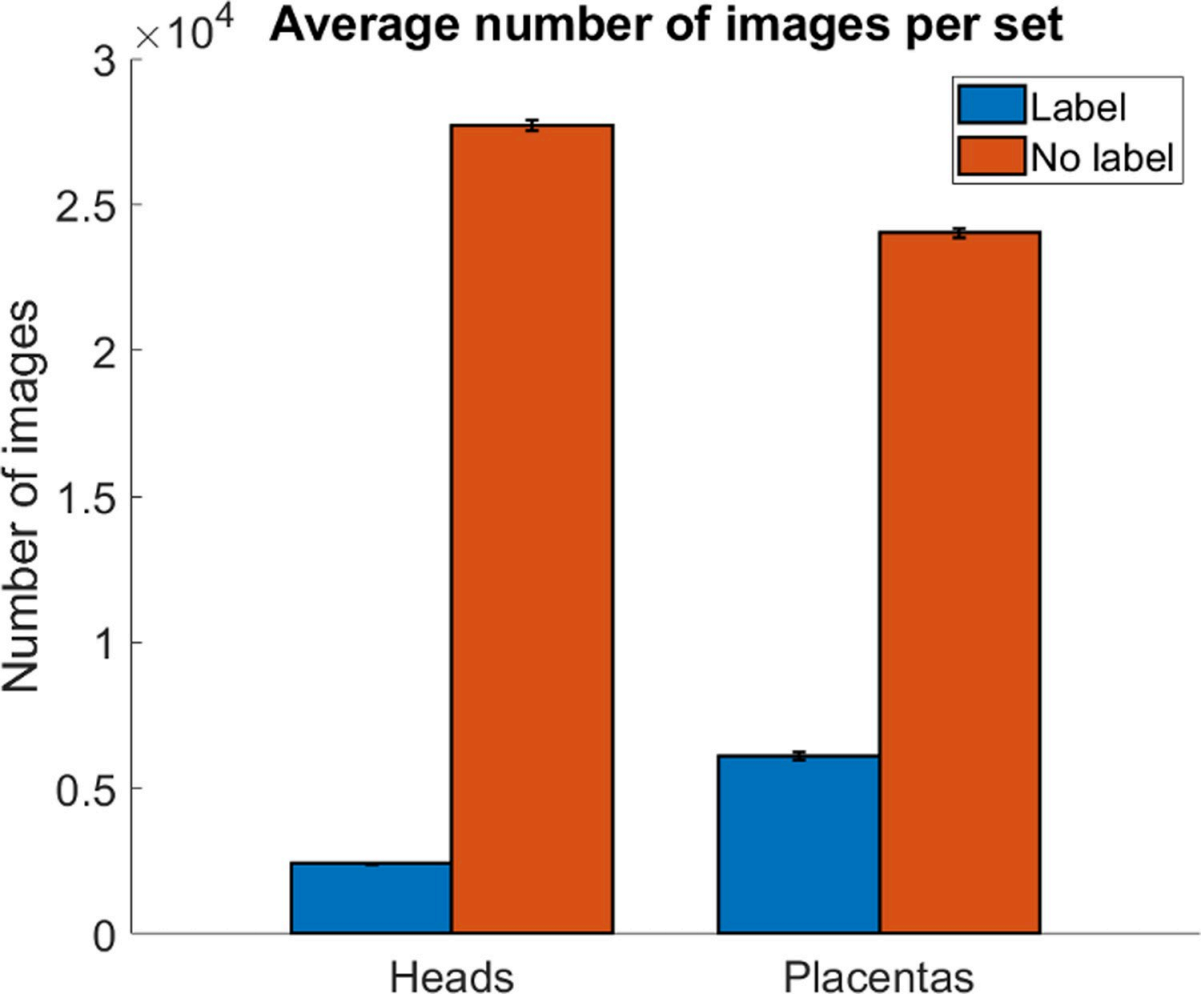
Image information contained in the training data set. The table shows the number of images labeled (blue) and without labels (orange). Bar plots indicate the average number of images for each classification. The error bar depicts the standard deviation between sets in leave-one-out cross-validation.

A leave-one-out approach was used to maximize the data available for evaluating the model. In this approach, the number of evaluations equals the number of patients. In this scheme, the learning model is applied once for each patient (data test), and the remaining patients are used for training and validation. With 30 segmented patients, the process was repeated 30 times, each time using a different patient as the single test case. This process is presented in detail in Table 1 and Fig 7. The training was performed in Keras 2.4.3 [25]. From the training data of a single set (29 patients), 80% of the frames with the structure of interest were randomly assigned for training and 20% of the frames with the structure of interest were randomly assigned for validation. The model received shuffled images for both training and testing.

**Table 1:** Set formation using leave-one-out cross-validation.

Set	Training data	Test data
1	Data from patient 2, 3, 4, …, 28, 29, 30	Data from patient 1
2	Data from patient 1, 3, 4, …, 28, 29, 30	Data from patient 2
3	Data from patient 1, 2, 4, …, 28, 29, 30	Data from patient 3
⋮	⋮	⋮
28	Data from patient 1, 2, 3, …, 27, 29, 30	Data from patient 28
29	Data from patient 1, 2, 3, …, 27, 28, 30	Data from patient 29
30	Data from patient 1, 2, 3, …, 27, 28, 29	Data from patient 30

**Fig 8:**
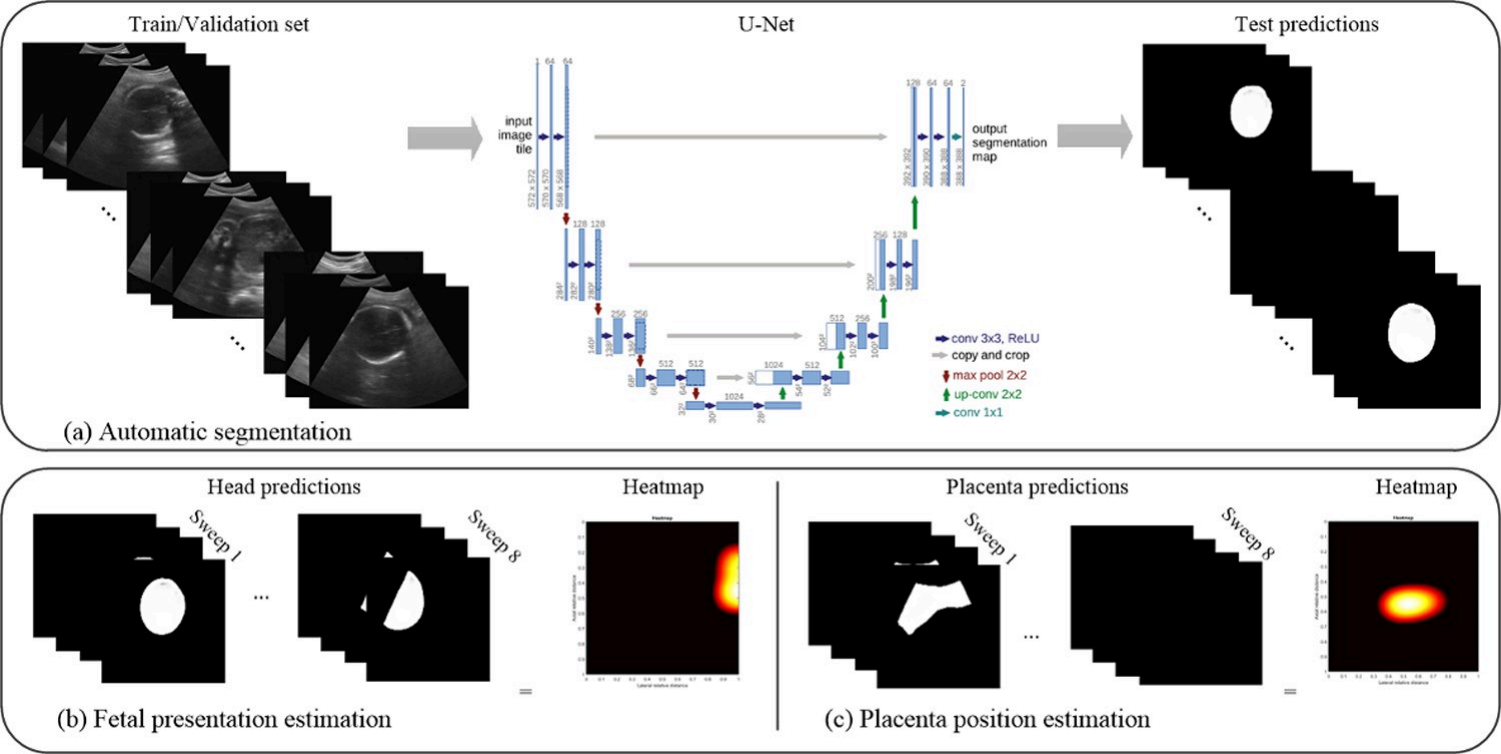
Overview of the proposed training method. (a) Automatic segmentation using the U-Net model [21]. (b) Fetal presentation prediction. (c) Placental location prediction.

This procedure aimed to segment the fetal head and placenta. The number of epochs for avoiding overfitting of head and placenta segmentation was 20 and 40, respectively. Fig 8 displays the flowchart of the training and testing stages for head segmentation.

#### Image post-processing

**Fig 9:**
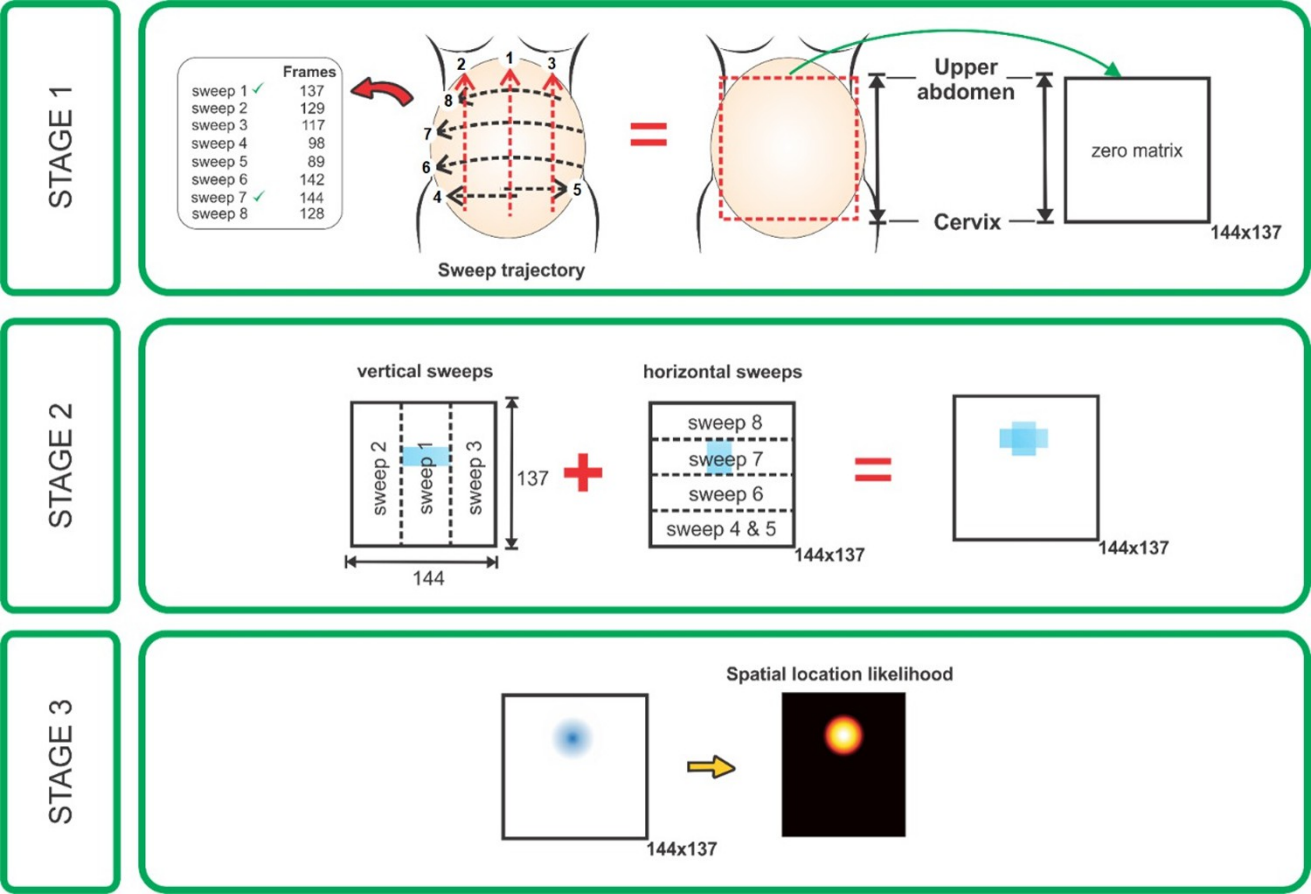
Scheme for the generation of the spatial location likelihood of the fetal head. Stage 1: The zero-matrix represents the pregnant abdomen, and it is formed considering the largest number of frames for horizontal and vertical sweeps. Sweeps with fewer frames are re-scaled to properly form the matrix. Stage 2: The frames containing the fetal head are colored. Stage 3: A Gaussian filter is applied only for representative purposes to finally produce the spatial location likelihood. Based on this map, the algorithm produces a diagnosis.

We combined predictions obtained from the U-Net model and the standardized nature of the VSI protocol to generate head and placental spatial location likelihood. The construction of the likelihood map was started by representing the uterine area in a 2D zero matrix. Then, based on the anatomical area covered by each sweep and the detection results, the likelihood map was progressively filled. Likelihood increased when frames from several sweeps overlapped in the same position. Fig 9 presents an example of the procedure for generating the spatial location likelihood of the fetal head. The size of the zero-matrix is dependent on the size of the largest horizontal and vertical sweeps. From the example shown in the figure, sweep 1 (a vertical sweep at midline from the maternal pelvis to upper abdomen) was formed by 137 ultrasound frames. Here, the fetal head was detected between frames 77 and 92. The resulting 2D zero matrix had 137 samples in the axial direction, and the elements between 77 and 92, indicated by the color block in the sweep direction, represent location of the fetal head. Similarly, sweep 7 (a horizontal sweep from maternal right flank to left flank at the level of the umbilicus) was formed by 144 ultrasound frames, with the color block indicating the location where the fetal head was detected along that sweep direction. A Gaussian filter was applied on the filled matrix, and finally, the spatial location likelihood was formed. The 2D map axis limits were normalized due to the abdominal area variation among all the participants. Fig 8 shows estimated masks of sweeps from head estimations used to generate a fetal presentation likelihood location map. Fig 9 represents the use of masks of the sweeps for estimating the 2D map of the placenta location. By using the eight sweeps acquired per patient, the localization of the placenta was then able to be determined.

### Hold-out test set

#### Fetal head and placental location

The leave-one-out cross-validation yielded prediction weights and detection/segmentation metrics for each one of the 30 evaluations. The prediction weights corresponding to the best metrics were then stored and used for future predictions. The 28 subsequently recruited patients were analyzed at this stage with the stored prediction weights, and the fetal head and placental location were detected for each one.

#### Biometric parameters

The U-Net model yielded prediction masks for each test patient. Automatic fetal head biometry was performed by fitting the predicted masks with an ellipse. The algorithm selected the largest estimated mask. The BPD diameter was calculated by multiplying two times the minor axis length. The HC was measured by calculating the border of the same selected mask.

#### Primary and secondary outcomes

Primary outcome variables were sensitivity, specificity, and agreement in fetal presentation (cephalic versus non-cephalic) and placental location (anterior, posterior, or fundal) by the U-Net model compared with the interpretations of the VSI acquisitions by an Obstetrician. Additional comparison was made to standard of care ultrasound separately obtained by a radiologist at the time of VSI examination.

This version of the Obstetric VSI protocol was not specifically designed for estimation of fetal biometry because the proper imaging planes needed for accurate and reproducible biometric measurements are not always present in the cine clips. Nonetheless, BPD and HC were chosen as exploratory outcome variables for testing diagnostic capacity of automated measurements of fetal biometry on VSI. Agreement and relative error of the U-Net model estimation of fetal head measurements (BPD and HC) were investigated as secondary outcome variables.

### Statistical analysis

Agreement on placental location and fetal presentation between physician assessment and model determination was assessed by overall agreement and Cohen’s κ. Resultant κ values were compared to a theoretical mean of 0 using a one-sample t-test. Biometry was compared between physician assessment and model determination using intraclass correlation coefficients (ICC) and Bland-Altman analysis. ICC values were calculated using a two-way mixed effects model for absolute agreement. Both ICC values and Bland-Altman bias were independently compared to a theoretical mean of 0 using a one-sample t-test. For κ and ICC values, 0–0.2 was defined as slight agreement, 0.21–0.4 as fair agreement, 0.41–0.6 as moderate agreement, 0.61–0.8 as substantial agreement, and 0.81–1 as almost perfect agreement [26]. All statistical analysis was performed using GraphPad PRISM (v6, GraphPad Software, Inc., San Diego, CA) and SPSS (v26, IBM Corporation, Armonk, NY).

## Results

### Participants

Fifty-eight third trimester pregnant women (mean gestational age 32.6 +/- 2.6 weeks) were studied. The women enrolled in the study were attending clinic for routine Obstetric follow-up and had no documented significant past medical history. Standard of care ultrasound revealed no developmental anomaly in any of the fetuses. The image diagnostic assessments assigned by the specialist to be ground truth for the U-Net model for the 30 patients used in the leave-one-out cross-validation and the 28 patients used in the hold-out testing phase are shown in Tables 3 and 4.

### Class imbalance

Our data had significant class imbalance with a minority of frames per patient containing fetal head or placenta. Preliminary tests of the model were executed by performing data augmentation to overcome class imbalance. Data augmentation attempts included brightness correction, image reflection (from left to right), blurring filter application, and contrast correction. As the images were acquired with a convex probe with a standardized region of interest, we did not attempt to perform image rotation. These attempts at data augmentation showed no significant improvement in our outcomes. An additional strategy was to delete images with no information instead of data augmentation. To do this we randomly deleted frames that did not contain fetal head to obtain a 1:2 ratio (from 1:10) and randomly deleted frames that did not contain placenta to obtain a 1:1 ratio (from 1:4). This again resulted in no improvement in metrics.

### Predicted diagnoses with leave-one-out cross-validation

**Fig 10:**
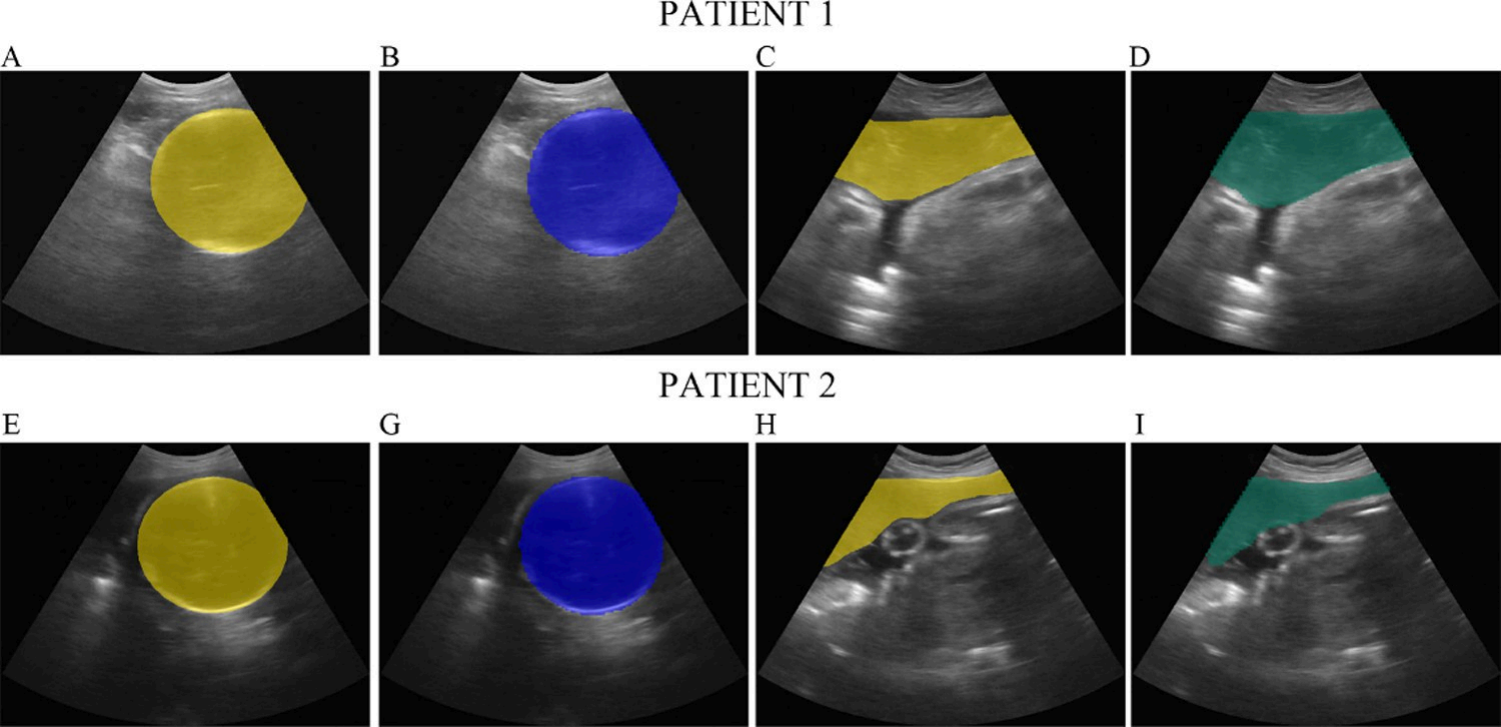
Ultrasound images. 2D ultrasound images from two representative patients (patient 1, upper row; patient 2, lower row) with overlaid color masks showing actual (yellow) versus predicted locations of the fetal head (blue) and placenta (green).

**Fig 11:**
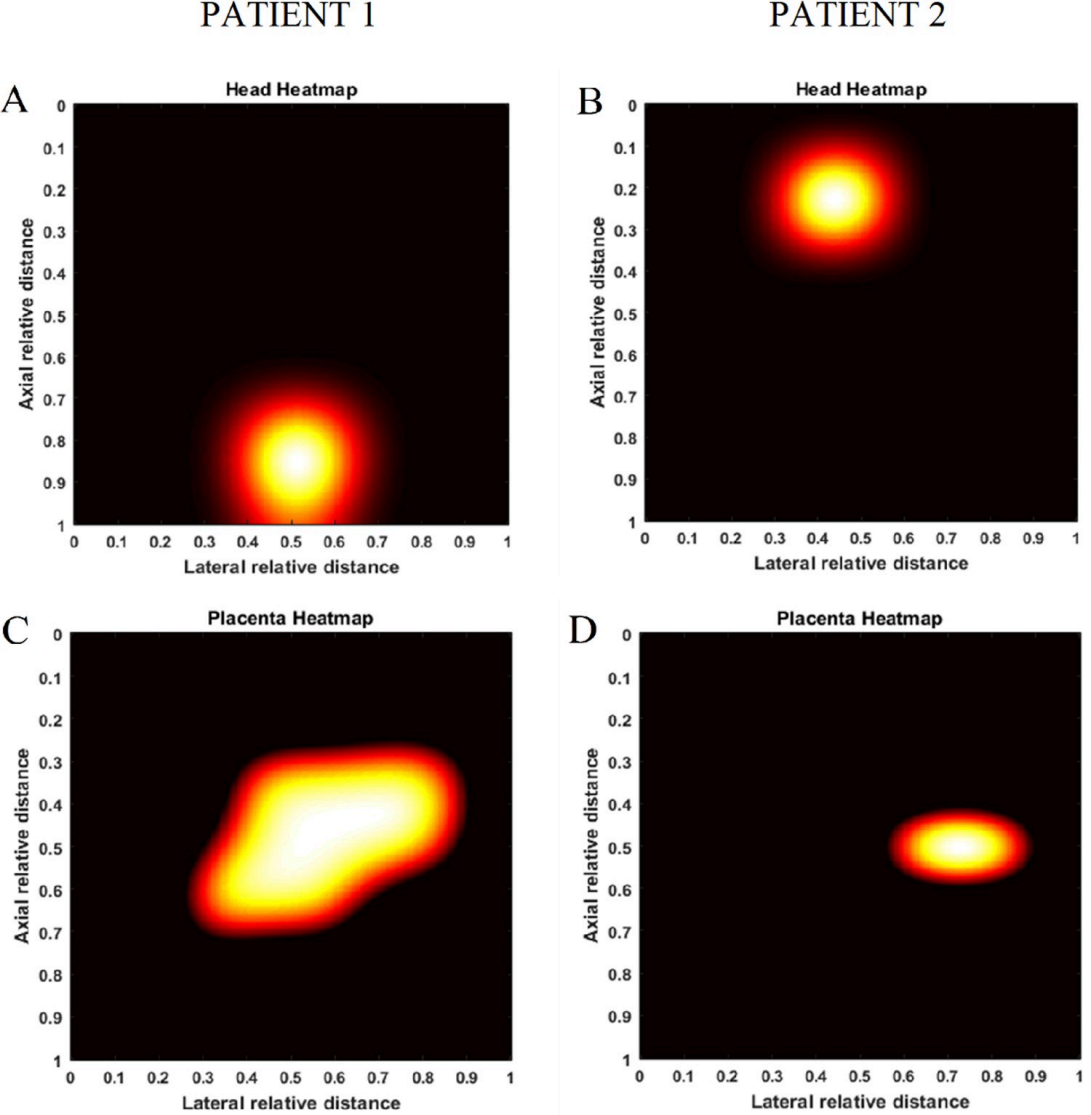
Heatmaps. Heatmaps of two representative patients showing the spatial locations of the fetal head and placenta. (A) Cephalic fetal presentation. (B) Non-cephalic fetal presentation. (C) Placenta anterior. (D) Placenta posterior.

2D ultrasound images with overlaid prediction masks of each region of interest are shown in Fig 10. The U-Net performance was evaluated considering the specialist’s manual segmentation as the ground truth. Table 2 shows the classification scores and metrics for fetal head and placenta. Representative 2D heat maps illustrating a cephalic versus non-cephalic fetus and anterior versus posterior placental location are seen in Fig 11. For the 30 subjects included in the leave-one-out cross-validation, our system yielded 100% agreement (κ = 1, p<0.0001) with the diagnostic assessment by a specialist for fetal presentation and 77% agreement (κ = 0.59, p<0.0001) for placental location (Table 3). When excluding cases where a fundal rating was used, agreement was 100% (κ = 1, p<0.0001). No cases of placenta previa were identified in either U-Net predictions or clinician assessment. The mean BPD and HC measurements predicted by the U-Net demonstrated low levels of relative error compared to specialist assigned measurements from VSI (5.6% and 7.9%, respectively, Table 4), with moderate to substantial agreement (BPD: ICC = 0.57; HC: ICC = 0.50). However, both predictions were significantly lower than physician measurements (BPD: Bias = -2.8 mm (-14.4–8.8 95% confidence interval (CI), p = 0.015); HC: Bias = -24.3 mm (-50.3–1.78 95% CI, p<0.0001)).

**Table 2:** Average detection and segmentation metrics for leave-one-out cross-validation.

Anatomic site	Detection					Segmentation		Number of images	
	Sensitivity	Specificity	Accuracy	PPV	NPV	Jaccard index	Area error (%)	Region of interest (ROI)	No ROI
Head	0.901	0.993	0.906	0.927	0.992	0.704	10.1	2402	27696
Placenta	0.733	0.950	0.80	0.80	0.946	0.862	25.6	6099	24014

**Table 3:** Comparison of qualitative diagnostic assessment of fetal presentation and placenta location assigned by an obstetrician from VSI versus automatic diagnosis.

Diagnostic parameter	Leave-one-out Cross-validation (n = 30)		Hold-out Test Set (n = 28)	
	Result from Obstetrician using VSI exam imaging	Result from U-Net model	Result from Obstetrician using VSI exam imaging*	Result from U-Net model
**Presentation**				
** Cephalic (n)**	25	25	26	26
** Non-cephalic (n)**	5	5	2	2
** Agreement (%)**	100		100	
** Sensitivity (%)**	100		100	
** Specificity (%)**	100		100	
** PPV (%)**	100		100	
** NPV (%)**	100		100	
**Placental location**				
** Anterior**	16	16	16	18
** Posterior**	13	10	8	5
** Fundal**	1	4	3	5
** Agreement (%)**	76.7		81.5	
** Sensitivity (%)**	87.5		100	
** Specificity (%)**	85.7		81.8	
** PPV (%)**	87.5		88.9	
** NPV (%)**	85.7		100	

*Obstetrician was unable to report placental position in one case in the hold-out test set which was ignored in calculations.

**Table 4:** Comparison of quantitative diagnostic assessment of fetal head circumference and biparietal diameter assigned by an obstetrician from VSI versus automatic diagnosis.

Diagnostic measurement	Leave-one-out Cross-validation (n = 30)				Hold-out Test Set (n = 28)			
	Result from Obstetrician using VSI exam imaging	Result from U-Net model	Relative error (%)	Bland-Altman Bias (95% CI, p value)	Result from Obstetrician using VSI exam imaging	Result from U-Net model	Relative error (%)	Bland-Altman Bias (95% CI, p value)
**Biparietal Diameter (mm)**	92.7±6.77	89.9 ± 6.73	5.6	-2.8	87.8 ± 6.42	87.1 ± 6.24	3.4	-0.71
				(-14.4–8.8, p = 0.015)				(-8.27–6.85, p = 0.34)
**Head circumference (mm)**	322±20.8	298 ± 22.1	7.7	-24.3	302 ± 22.5	286 ± 19.2	5.6	-15.8
				(-50.3–1.78, p<0.0001)				(-38.6–7.14, p<0.0001)

Bias is defined as value assigned by U-Net model–assigned by specialist.

### Predicted diagnoses in the hold-out test set

As a result of the prior analysis, 30 models for the head and 30 models for the placenta were obtained (one model per set evaluated, each model with its corresponding trainable parameters stored in a weights file). We selected the models for the placenta and head with the best combination of evaluation parameters: sensitivity, specificity, positive predictive value (PPV), negative predictive value (NPV), Jaccard index, pixel accuracy, and segmentation area error. In order to determine the diagnostic capability of the proposed method, 28 unique new patients were analyzed using these models. The agreement between the model and specialist’s assessment for fetal presentation was 100% (Table 3). The agreement for placental location was 81.5% (κ = 0.5, p<0.0001) with sensitivity and specificity of 100% and 81.8%, respectively. When considering only anterior/posterior locations (excluding fundal), agreement was 90.9% (κ = 0.74, p = 0.002), with 100% sensitivity and 66.7% specificity. No cases of placenta previa were identified by U-Net or clinician assessment.

The relative error of the estimated BPD and HC measurements by the U-Net model were 3.4% and 5.6%, respectively (Table 4), with substantial to almost perfect agreement (BPD: ICC = 0.82; HC: ICC = 0.66). U-Net predictions were again lower than physician measurements for HC (Bias = -15.8 (-38.6–7.14 95% CI, p<0.0001)), though results were similar to each other for BPD (Bias = -0.71 (-8.27–6.85 95% CI, p = 0.34)). These results are overall similar to those obtained in the previous tests, which sheds light on the robustness of the method proposed for the automatic evaluation of novel patients.

### Comparison to standard of care ultrasound

The automatic detection of placental location, fetal position, and biometry measurements assigned by the U-Net also showed good agreement with standard of care ultrasound (performed and interpreted by a radiologist) (Tables 5 and 6). During leave-one-out cross-validation, there was 90.4% agreement for placental position when examinations with fundal position were excluded (Cohen’s κ 0.77, p = 0.002) with 93% sensitivity and 83% specificity for anterior placenta position. In the hold-out test set, there was 90% agreement for placental position when examinations with fundal position were excluded (Cohen’s κ 0.74, p = 0.003) with 100% sensitivity and 66.7% specificity for anterior placenta position. Standard of care ultrasound and U-Net both identified no cases of placenta previa. Agreement on fetal position during leave-one-out cross-validation was 97% (Cohen’s κ 0.84, p = 0.001) with 96% sensitivity and 100% specificity for the vertex position. Agreement on fetal presentation in the hold-out test set was 96.2% (Cohen’s κ 0.65, p = 0.08) with 96% sensitivity and 100% specificity for vertex position. During leave-one-out cross-validation there was 9.4% relative error for BPD and 6.6% relative error for HC. In the hold-out test set there was 8.4% relative error for BPD and 3.7% relative error for HC. S1 and S2 Tables include supplemental analysis for comparison of VSI and standard of care ultrasound results. The comparison between VSI and standard of care has been previously studied [8].

**Table 5:** Comparison of qualitative diagnostic assessment of fetal presentation and placenta location assigned by a radiologist from standard of care imaging versus automatic diagnosis.

Diagnostic parameter	Leave-one-out Cross-validation (n = 30)		Hold-out Test Set (n = 28)	
	Result from radiologist using standard of care imaging*	Result from U-Net model	Result from radiologist using standard of care imaging*	Result from U-Net model
**Presentation**				
** Cephalic (n)**	26	25	25	26
** Non-cephalic (n)**	3	5	1	2
** Agreement (%)**	96.6%		96.2%	
** Sensitivity (%)**	96.2%		96.0%	
** Specificity (%)**	100%		100%	
** PPV (%)**	100%		100%	
** NPV (%)**	75.0%		50.0%	
**Placental location**				
** Anterior**	15	16	14	18
** Posterior**	9	10	10	5
** Fundal**	6	4	4	5
** Agreement (%)**	66.7%		71.4%	
** Sensitivity (%)**	93.3%		100%	
** Specificity (%)**	86.7%		71.4%	
** PPV (%)**	87.5%		77.8%	
** NPV (%)**	92.9%		100%	

*Radiologist did not report the fetal position in 1 case during leave-one-out cross-validation and in 2 cases in the hold-out test set which were ignored in calculations.

**Table 6:** Comparison of quantitative diagnostic assessment of fetal head circumference and biparietal diameter assigned by a radiologist from standard of care imaging versus automatic diagnosis.

Diagnostic measurement	Leave-one-out Cross-validation (n = 30)				Hold-out Test Set (n = 28)			
	Result from radiologist using standard of care imaging	Result from U-Net model	Relative error (%)	Bland-Altman Bias (95% CI, p value)	Assigned from radiologist using standard of care imaging	Result from U-Net model	Relative error (%)	Bland-Altman Bias (95% CI, p value)
**Biparietal Diameter (mm)**	83.3±8.46	89.9±6.73	9.4%	6.64 (-7.65–20.9, p<0.0001)	80.5±6.59	87.1±6.24	8.4%	6.57 (-1.44–14.6, p<0.0001)
**Head circumference (mm)**	300±26.9	298±22.1	6.6%	-1.87 (-53.5–49.8, p = 0.70)	289.8±20.3	286±19.2	3.7%	-3.62 (-29.7–22.5, p = 0.16)

Bland-Altman bias is defined as value assigned by specialist (VSI)–assigned by radiologist (SOC)

### Gestational age analysis

Analysis of gestational age calculations are shown in Table 7. U-Net and VSI showed significant differences in gestational age in both leave-one-out cross-validation and the hold-out test set. In leave-one-out cross-validation, U-Net predicted an estimated gestational age of 234±19.2 days compared to VSI which showed an estimated gestational age of 252±19.6 days (p <0.0001). In the hold-out test set, U-Net predicated an estimated gestational age of 225±16.5 days compared to VSI which showed an estimated gestational age of 235±19.1 days (p <0.0001). In the analysis of U-Net compared to standard of care dating, estimated gestational age calculated from these measures were not significantly different between U-Net and standard of care ultrasound. In leave-one-out cross-validation, U-Net predicted an estimated gestational age of 234±19.2 days compared to standard of care which showed an estimated gestational age of 229±22.6 days (p = 0.22). In the hold-out test set, U-Net predicated an estimated gestational age of 225±16.5 days compared to standard of care which showed an estimated gestational age of 223±19.9 days (p = 0.5).

**Table 7:** Analysis of gestational age accuracy across U-Net, VSI, and standard of care imaging.

Comparison	Data Set	Within 7 days	Within 14 days
U-Net and VSI	Leave-one-out	16.7%	30.0%
	Hold-out	28.6%	71.4%
U-Net and SOC	Leave-one-out	40.0%	70.0%
	Hold-out	64.3%	89.3%
VSI and SOC	Leave-one-out	13.3%	36.7%
	Hold-out	28.6%	60.7%

## Discussion

The majority of the world lacks access to ultrasound diagnostic imaging, a vital component of high-quality Obstetric care. Obstacles to deploying Obstetric ultrasound in rural and under-resourced areas relate to both limited trained healthcare personnel and infrastructure, such as costly devices, and limitations of telemedicine such as high-speed internet. Through the integration of VSI and U-Net, this automatic model for detecting fetal position, placental position, and fetal biometry offers a way to overcome resource limitations such as the lack of sonographers, radiologists, and high-speed internet (Fig 4). Furthermore, this approach can allow rapid interpretation of imaging findings when a delay may adversely affect patient outcomes. Therefore, this approach offers substantial promise in improving access to ultrasound in rural areas.

In this study, there was 100% agreement between our automatic model’s prediction and a specialist’s diagnostic assessment of fetal presentation when compared to VSI protocol and 96% agreement when compared to standard of care ultrasound. The discrepancy with standard of care ultrasound may be attributed to dynamic fetal position between the standard of care ultrasound and VSI exam. In general, false detection of fetal position could arise from the fetal head not being in the optimal plane for analysis for enough frames. Clinically, these results suggest this system has potential for accurate automatic detection of non-cephalic fetuses near the time of delivery, which would allow for referral to a higher level of care for delivery attended by trained healthcare providers.

Prediction of placental location by the automatic model showed 76.7% agreement when compared to the VSI protocol, although this improved to 100% when the category of “fundal” was removed and anterior versus posterior location were directly compared. In practice, fundal position overlaps with both anterior and posterior placental positions, confounding analysis of the agreement. In clinical practice, the main concern is to identify placenta previa. While we had no cases of placenta previa to analyze, given the observed agreement in placental position, the system would theoretically detect this positioning as well. Even though the system was not “trained” with cases of placenta previa, presumably if a case of placenta previa was introduced, the placenta would be identified and mapped to the lowest part of the 2D matrix.

The placental structure is irregular and the echogenicity uneven, making training of the model challenging. In addition, the placental masks delineated by the specialist often spanned several frames in the cine clips. The model was trained by shuffling data, which may alter continuity through frames. This contributed to the challenges in using the prediction model to identify placental location. In the future, splitting the videos into several shorter clips for the data training (instead of individual frames) can maintain continuity and may improve network identification of placental location. The false detection of placenta location when excluding the overlapping fundal position we observed may be due to the complexity of mapping the 3D placental structure on the 2D matrix. Clinically, the use of automatic detection of placental location could allow for identification of low-lying placenta or placenta previa, which is associated with maternal hemorrhage and death if undiagnosed prior to delivery. If the model detects a placenta in the lower uterine body or near the cervix, patients could be referred for ultrasound to confirm these suspected findings. In the current study, several patients had a non-cephalic presentation, but there were no patients with pathologic location of the placenta (placenta previa or low-lying near the internal cervical os) which is not surprising since the incidence of placenta previa has been estimated to be 5 in 1000 pregnancies [27]. Future studies are necessary to confirm our findings in an enriched population of subjects with a higher prevalence of abnormal ultrasound findings, such as transverse/breech fetal lie or placenta previa.

We observed generally higher agreement between U-Net and VSI and standard of care ultrasound in our hold-out test set. We hypothesize that this relates to data characteristics of the hold-out sample. It may be that the planes needed to optimally locate the structures of interest in the hold-out set were more often obtained due to random chance. Interestingly, U-Net performed better than VSI when predicting fetal age when compared to standard of care. This phenomenon may be due to miscalibration of the VSI teleultrasound system viewer. Previous studies have shown VSI to over measure in both Obstetric and thyroid ultrasound [8, 20].

Automated detection for measurement of fetal biometry demonstrated low levels of relative error for HC and BPD measurements by the U-Net model compared to both VSI and standard of care ultrasound. The are several possible reasons for differences in measurements from U-net compared to our reference standards. Human error and differences in the plane the structure of interest was measured are two possibilities. In addition, VSI has been shown to overestimate measures which may be due to slight miscalibration of the system [8, 20]. Because images are acquired by non-specialists using a standard protocol, fetal biometry planes to measure these structures may not continually be optimized in the video clips obtained. Prior study has demonstrated that even when non-standard image planes (closely approximating but not completely fulfilling standard imaging guidelines) are used to estimate fetal biometry measurements from VSI scans, there is no significant difference in gross estimations of fetal size [8]. In our experience, fetal head is the most likely structure to be oriented in the correct plane for measurement when the standard sagittal or transverse sweeps across the maternal abdomen are performed in the VSI protocol, and therefore these measurements were chosen as exploratory outcomes for this study. Further investigation is needed to determine the utility of automated detection of the abdominal circumference or femur length by the U-Net model. These measurements are more challenging to obtain as they are more commonly oriented in oblique planes outside the standard sweeps of the VSI protocol. Despite this, clinically, the use of fetal head biometry alone for gross estimation of fetal size may be useful to identify pregnancies which need referral to a higher level of care for ultrasound with a trained sonographer and specialist to diagnose fetal growth abnormalities. Future studies could investigate the model’s predictive capabilities for detecting femur length and abdominal circumference (for a complete fetal biometry evaluation), as well as additional diagnostic tasks such as fetal heart rate or additional fetal anatomic structures.

Ultrasound segmentation is challenging because it depends on the image quality [28]. The VSI scans in this study were completed on a portable ultrasound machine with relatively low resolution. Fetal features were sometimes hard to distinguish, even by the specialist performing the diagnostic assessment. In the context of these limitations, these results of the automatic model predictions are especially promising. A limitation of the current model methodology is that U-Net requires ultrasound image size reduction for leave-one-out cross-validation. This stage may alter the resolution of fetal structures and disturb the capability of the network to segment the structures accurately. Enlarging both the contracting and expansive paths may overcome this drawback. However, a larger image size would demand more graphics processing unit memory. Convolutional neural networks have a computational cost for training. This network can be improved by adding a generative adversarial network (GAN) to help the learning process. The GAN model has a generator and discriminator modules [29]. The generator module can focus on the segmentation part, followed by the discriminator module, comparing the segmentation with the ground truth.

There are profound health and healthcare inequalities between countries. The global distribution of poor antenatal care across countries contributes to radically different health outcomes for mothers and children across the globe [30–33]. Unfortunately, there are many challenges to delivering healthcare to these rural and under-resourced areas. “Duffle bag medicine” and medical tourism offer temporary attempts to address medical needs but do not offer long-term responsibility, accountability, collaboration with local stakeholders, cultural sensitivity, or a goal to reinforce the local medical infrastructure in a sustainable way [34]. Deploying innovative technologies and practices to reduce maternal and perinatal morbidity and mortality can only be successful if implemented and disseminated within the capabilities of existing local public health care structures and with consideration of available resources [35]. The potential impact on short- and long-term health outcomes extends to the public health sector. Poor health infant outcomes put the infant at risk for social and behavioral issues (e.g., physical and learning disabilities), and place new demands on the mother, family and community. These could include impact on household income, support to other children and additional requirements for services and support. Maternal morbidity and mortality impact the family and community in similar ways.

This automatic diagnostic model shows potential to predict fetal presentation, placental location, and fetal biometry from ultrasound images using an approach that eliminates the need for trained sonographers, specialists, costly equipment, and high-speed internet. In this study, there was excellent agreement with this automatic approach and expert interpretation from a physician specialist. Obstetric VSI combined with the U-Net model predictions therefore offers a potential new horizon in sustainably expanding vital Obstetric ultrasound imaging in rural areas.

## Supporting information

S1 Table Comparison of qualitative diagnostic assessment of fetal presentation and placenta location assigned by an obstetrician from VSI versus standard of care imaging. (DOCX)

S2 Table Comparison of quantitative diagnostic assessment of fetal head circumference and biparietal diameter assigned by an obstetrician from VSI versus standard of care imaging. (DOCX)
