# Supplementary material for: No sonographer, no radiologist: New system for automatic prenatal detection of fetal biometry, fetal presentation, and placental location
Source: PLoS One. 2022 Feb 9;17(2):e0262107. doi: 10.1371/journal.pone.0262107 (PMC8827457; doi:10.1371/journal.pone.0262107)
Supplement: S1 Table — (DOCX) [file pone.0262107.s001.docx]

**Comparison of qualitative diagnostic assessment of fetal presentation and placenta location assigned by an Obstetrician from VSI versus standard of care imaging.**

| **Diagnostic parameter** | **Leave-one-out Cross-validation (n=30)** | | **Hold-out Test Set (n=28)** | |
| --- | --- | --- | --- | --- |
|  | Result from radiologist using standard of care imaging* | Result from Obstetrician using VSI exam imaging* | Result from radiologist using standard of care imaging* | Result from Obstetrician using VSI exam imaging* |
| **Presentation** | | | | |
| **Cephalic (n)** | 26 | 25 | 25 | 26 |
| **Non-cephalic (n)** | 3 | 5 | 1 | 2 |
| **Agreement (%)** | 96.6% | | 96.1% | |
| **Sensitivity (%)** | 96.2% | | 96.0% | |
| **Specificity (%)** | 100% | | 100% | |
| **PPV (%)** | 100% | | 100% | |
| **NPV (%)** | 75.0% | | 50.0% | |
| **Placental location** | | | | |
| **Anterior** | 15 | 16 | 14 | 16 |
| **Posterior** | 9 | 13 | 10 | 8 |
| **Fundal** | 6 | 1 | 4 | 3 |
| **Agreement (%)** | 76.7% | | 77.8% | |
| **Sensitivity (%)** | 100% | | 100% | |
| **Specificity (%)** | 93.3% | | 84.6% | |
| **PPV (%)** | 93.8% | | 87.5% | |
| **NPV (%)** | 100% | | 100% | |

*Radiologist did not report 1 fetal positioning in leave-one-out cross-validation and 2 cases in the hold-out test set which were ignored in calculations. Obstetrician was unable to report placental position during the experimental phase in one case which was ignored in calculations.
