# Supplementary material for: No sonographer, no radiologist: New system for automatic prenatal detection of fetal biometry, fetal presentation, and placental location
Source: PLoS One. 2022 Feb 9;17(2):e0262107. doi: 10.1371/journal.pone.0262107 (PMC8827457; doi:10.1371/journal.pone.0262107)
Supplement: S2 Table — (DOCX) [file pone.0262107.s002.docx]

**Comparison of quantitative diagnostic assessment of fetal head circumference and biparietal diameter assigned by an Obstetrician from VSI versus standard of care imaging.**

| **Diagnostic measurement** | **Leave-one-out Cross-validation (n=30)** | | | | **Hold-out Test Set (n=28)** | | | |
| --- | --- | --- | --- | --- | --- | --- | --- | --- |
|  | Result from radiologist using standard of care imaging | Result from Obstetrician using VSI | Relative error (%) | Bland-Altman Bias (95% CI,  p value) | Result from radiologist using standard of care imaging | Result from Obstetrician using VSI | Relative error (%) | Bland-Altman Bias (95% CI,  p value) |
| **Biparietal Diameter (mm)** | 83.3±8.46 | 92.7±6.77 | 13.7% | 9.45 (-6.9-25.8, p<0.0001) | 80.5±6.59 | 87.8±6.42 | 8.4% | 7.28 (-0.57-15.1, p<0.0001) |
| **Head circumference (mm)** | 300±26.9 | 322±20.8 | 9.0% | 22.4 (-26.0-70.8, p<0.0001) | 289.8±20.3 | 302±22.5 | 4.7% | 12.1 (-16.1-40.3, p=0.0001) |

Bland-Altman bias is defined as value assigned by specialist (VSI) – assigned by radiologist (SOC)
